# Communicative-Pragmatic Assessment Is Sensitive and Time-Effective in Measuring the Outcome of Aphasia Therapy

**DOI:** 10.3389/fnhum.2017.00223

**Published:** 2017-05-19

**Authors:** Benjamin Stahl, Bettina Mohr, Felix R. Dreyer, Guglielmo Lucchese, Friedemann Pulvermüller

**Affiliations:** ^1^Department of Neurology, Charité Universitätsmedizin Berlin, Campus MitteBerlin, Germany; ^2^Max Planck Institute for Human Cognitive and Brain SciencesLeipzig, Germany; ^3^Department of Neurology, Universitätsmedizin GreifswaldGreifswald, Germany; ^4^Brain Language Laboratory, Department of Philosophy and Humanities, Freie Universität BerlinBerlin, Germany; ^5^Department of Psychiatry, Charité Universitätsmedizin Berlin, Campus Benjamin FranklinBerlin, Germany; ^6^Berlin School of Mind and Brain, Humboldt-Universität zu BerlinBerlin, Germany

**Keywords:** aphasia, Intensive Language-Action Therapy (ILAT), Constraint-Induced Aphasia Therapy (CIAT), communicative-pragmatic speech-language testing, neuroscience of pragmatics, formulaic language

## Abstract

A range of methods in clinical research aim to assess treatment-induced progress in aphasia therapy. Here, we used a crossover randomized controlled design to compare the suitability of utterance-centered and dialogue-sensitive outcome measures in speech-language testing. Fourteen individuals with post-stroke chronic non-fluent aphasia each received two types of intensive training in counterbalanced order: conventional confrontation naming, and communicative-pragmatic speech-language therapy (Intensive Language-Action Therapy, an expanded version of Constraint-Induced Aphasia Therapy). Motivated by linguistic-pragmatic theory and neuroscience data, our dependent variables included a newly created diagnostic instrument, the Action Communication Test (ACT). This diagnostic instrument requires patients to produce target words in two conditions: (i) utterance-centered object naming, and (ii) communicative-pragmatic social interaction based on verbal requests. In addition, we administered a standardized aphasia test battery, the Aachen Aphasia Test (AAT). Composite scores on the ACT and the AAT revealed similar patterns of changes in language performance over time, irrespective of the treatment applied. Changes in language performance were relatively consistent with the AAT results also when considering both ACT subscales separately from each other. However, only the ACT subscale evaluating verbal requests proved to be successful in distinguishing between different types of training in our patient sample. Critically, testing duration was substantially shorter for the entire ACT (10–20 min) than for the AAT (60–90 min). Taken together, the current findings suggest that communicative-pragmatic methods in speech-language testing provide a sensitive and time-effective measure to determine the outcome of aphasia therapy.

## Introduction

More than a decade ago, clinical research has demonstrated the efficacy of intensive speech-language therapy (SLT) in neurological patients (Bhogal et al., 2003; Cherney et al., 2008; Brady et al., 2016). Most notably, a series of randomized controlled trials (RCTs) confirmed the short- and long-term benefit from Intensive Language-Action Therapy (ILAT, an extended form of Constraint-Induced Aphasia Therapy) in post-stroke chronic non-fluent aphasia (e.g., Pulvermüller et al., 2001; Meinzer et al., 2005, 2007; Berthier et al., 2009; Szaflarski et al., 2015). However, little is known about the relative adequacy of current methods used to evaluate the outcome of SLT. Existing methods generally fall into two different categories. On the one hand, utterance-centered aphasia test batteries focus, by definition, on isolated skills in verbal expression, including the ability to name objects, describe scenes or repeat words and sentences, regardless of their communicative function (e.g., Goodglass and Kaplan, 1972; Kertesz, 1982; Huber et al., 1984). On the other hand, dialogue-sensitive diagnostic instruments in SLT aim to assess the proficiency level in everyday communication based on role playing (e.g., Holland, 1980; Blomert et al., 1994) or on questionnaires with ratings by clinicians and family members (e.g., Lomas et al., 1989; Pulvermüller and Berthier, 2008). Considering the importance of appropriate outcome measures in aphasia therapy, surprisingly few attempts have been made to directly compare the practicability of utterance-centered and dialogue-sensitive methods in speech-language testing. The present work seeks to address this issue.

Consistent with the notion that the primary function of language emerges from social interaction (Wittgenstein, 1953; Bruner, 1975; Tomasello, 2005), linguistic-pragmatic theory implies that, compared to utterance-centered approaches, dialogue-sensitive diagnostic instruments cover a wider range of aspects observed in everyday communication (Austin, 1962; Searle, 1969; Horn and Ward, 2008). As one example, verbal requests differ from object naming in that they entail a richer action-sequence structure, associated “common ground” and theory of mind about assumptions and intentions of the conversation partner. Accordingly, a growing body of neuroscience data shows that making verbal requests elicits stronger cortical language and motor responses than object naming performed with the same linguistic materials (Egorova et al., 2013, 2014, 2016). Further neuroscience data indicate that the neural bases of language and action are functionally interlinked (Pulvermüller et al., 2005; Glenberg et al., 2008; Kemmerer et al., 2008; Willems et al., 2011; Andres et al., 2015). It has therefore been proposed that providing context of communication and social interaction facilitates language processing (Berthier and Pulvermüller, 2011), a claim recently supported by RCT evidence in persons with chronic non-fluent aphasia (Stahl et al., 2016). Consequently, a thorough analysis of verbal expression skills may require more than utterance-centered speech-language testing where patients produce words or sentences in artificial, often school-like settings (e.g., “What do you *see*?”—“A bottle.”). Instead, the validity and reliability of any such analysis may improve in dialogue-sensitive speech-language testing where patients engage in communication and social interaction (e.g., “What do you *want*?”—“The bottle.”).

To investigate the practicability of utterance-centered and dialogue-sensitive outcome measures in speech-language testing, we conducted a pilot study using a crossover randomized controlled design. Individuals with post-stroke chronic non-fluent aphasia each received two types of intensive training in counterbalanced order: conventional confrontation naming (Naming Therapy), and communicative-pragmatic SLT (ILAT). Patients underwent speech-language testing before and immediately after each type of training. Along with a standardized aphasia test battery, our outcome measures included a newly created diagnostic instrument focusing on (i) utterance-centered object naming, and (ii) communicative-pragmatic social interaction based on verbal requests. As summarized above, linguistic-pragmatic theory and neuroscience data suggest that verbal requests might be especially suited to evaluate the outcome of SLT, given their distinct action-sequence structure and relevance to everyday life.

## Materials and methods

### Participants

Fourteen persons with post-stroke chronic non-fluent aphasia were recruited, screened and agreed to participate in the current study. All patients were native speakers of German who had not received intensive SLT in the year prior to inclusion in the study. Patients were aged 32–73 years (mean age: 50 years; standard deviation: 12 years) and right-handed before stroke according to the Edinburgh Handedness Inventory (Oldfield, 1971). The trial excluded individuals with severe cognitive disorders that may have caused problems during therapy or testing. To prevent non-treatment effects resulting from spontaneous remission, patients were at least one year post-onset of stroke at the time of initial testing. The study was registered prospectively (URL: www.germanctr.de; identifier: DRKS00005482) and approved by the ethics review board at the Charité Universitätsmedizin Berlin, Campus Benjamin Franklin, Germany (reference number: EA4/122/12), with written informed consent obtained from all patients.^1^

**Figure 1:**
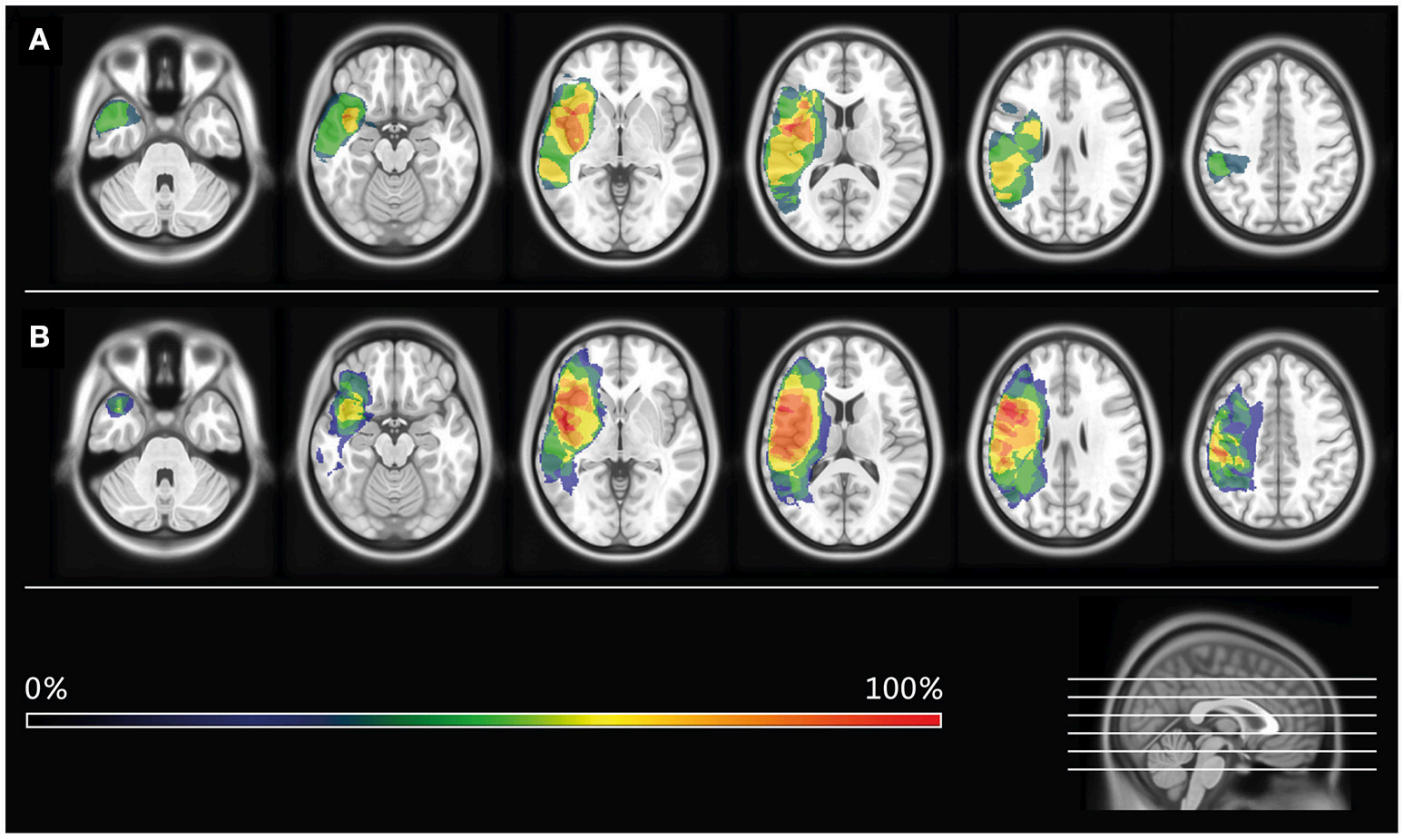
**Lesion overlay maps**. Lesion overlay maps of patients receiving Intensive Language-Action Therapy prior to Naming Therapy (Group I; see **A**), or vice versa (Group II; see **B**). Different colors refer to the degree of lesion overlap in each treatment group.

The diagnosis of aphasia was confirmed in each patient using a standardized aphasia test battery, the Aachen Aphasia Test (AAT; Huber et al., 1984). Focusing on non-verbal short-term memory, our patient sample scored, on average, within the normal range on the Corsi Block-Tapping Task (Kessels et al., 2000). Structural T_1_-weighted magnetic resonance imaging was performed using a 3T Magnetom Trio scanner (Siemens Medical Solutions, Erlangen, Germany). All patients had suffered a single cerebrovascular accident with subsequent lesions in parts of the left frontal, parietal, and temporal lobes, as well as in adjacent subcortical areas. Two clinical neuroscientists manually delineated and superimposed the precise locations of lesioned voxels in all patients using the software MRIcron (Rorden and Brett, 2000; for lesion overlay maps, see Figure 1; for individual case histories and baseline test scores, see Tables 1, 2).

**Table 1:** **Patient histories**.

**Patient**	**Gender**	**Age (in years)**	**Education level (in years)**	**Months after onset of disease**	**Origin**
01	Female	41	18	97	Left MCA ischemia
02	Male	49	14	52	Left MCA ischemia
03	Male	54	21	49	Left MCA ischemia
04	Female	35	12	13	Left MCA ischemia
05	Male	32	14	40	Left MCA ischemia
06	Male	62	17	23	Left MCA ischemia
Group I: Mean (SD)		45.5 (10.6)	16.0 (3.0)	45.7 (26.8)	
07	Male	73	19	61	Left MCA ischemia
08	Female	39	12	78	Left MCA ischemia
09	Female	49	13	149	Left MCA ischemia
10	Male	51	12	42	Left MCA ischemia
11	Male	63	13	31	Left MCA ischemia
12	Female	47	12	245	Left MCA ischemia
13	Female	37	11	30	Left MCA ischemia
14	Male	65	25	239	Left MCA ischemia
Group II: Mean (SD)		53.3 (11.9)	14.6 (4.6)	109.4 (84.5)	

*Patients are listed according to treatment order: Group I (Intensive Language-Action Therapy; Naming Therapy), and Group II (Naming Therapy; Intensive Language-Action Therapy)*.

*MCA, Middle cerebral artery; SD, Standard deviation*.

**Table 2:** **Baseline test scores**.

**Patient**	**Token Test**	**Repetition**	**Naming**	**Comprehension**	**CBTT**	**Diagnosis**
01	51	59	53	70	6	Mild Broca's aphasia
02	51	61	53	64	7	Mild Broca's aphasia
03	48	45	56	62	6	Mild-moderate Broca's aphasia
04	33	37	39	47	7	Severe Broca's aphasia
05	56	54	57	78	7	Mild Broca's aphasia
06	42	43	39	47	5	Severe global aphasia
Group I: Mean (SD)	46.8 (7.5)	49.8 (8.8)	49.5 (7.6)	58.3 (16.3)	6.3 (0.7)	
07	41	42	41	34	3	Severe global aphasia
08	44	46	47	45	6	Moderate Broca's aphasia
09	48	48	48	53	4	Moderate Broca's aphasia
10	51	45	49	49	6	Moderate Broca's aphasia
11	54	52	49	48	6	Moderate Broca's aphasia
12	55	59	68	62	6	Mild Broca's aphasia
13	54	53	53	57	6	Mild-moderate Broca's aphasia
14	47	52	46	49	6	Moderate Broca's aphasia
Group II: Mean (SD)	49.3 (4.8)	49.6 (5.1)	50.1 (7.5)	49.5 (8.9)	5.4 (1.1)	

*Individual t-scores obtained on the Aachen Aphasia Test (Huber et al., 1984). Token Test: severe (0–43), moderate (44–53), light (54–62) or mild disorder (≥63). Repetition: severe (0–43), moderate (44–53), light (54–62) or mild disorder (≥63). Naming: severe (0–43), moderate (44–53), light (54–62) or mild disorder (≥63). Comprehension: severe (0–43), moderate (44–53), light (54–63) or mild disorder (≥64). Non-verbal short-term memory was assessed using the Corsi Block-Tapping Task (Kessels et al., 2000). All scores and means are shown separately for Group I (Intensive Language-Action Therapy; Naming Therapy), and Group II (Naming Therapy; Intensive Language-Action Therapy)*.

*CBTT, Corsi Block-Tapping Task; SD, Standard deviation*.

### Study design and randomization

In a crossover design, patients were randomly assigned to one of two treatment orders: ILAT administered prior to Naming Therapy (Group I; *n* = 6), or vice versa (Group II; *n* = 8). The group allocation was consistent with a previously determined computer-generated series of random numbers. Mann-Whitney *U* tests suggested that this randomization procedure did not lead to significant differences between Group I and Group II with regard to: age, education level, months after onset of disease, aphasia test scores at baseline, non-verbal short-term memory, individual lesion size, and weekly hours of SLT before inclusion in the study. Since patients with aphasia usually suffer from concomitant deficits in motor planning, it is important to note that Group I and Group II were similarly affected by apraxia of speech, as diagnosed by two clinical linguists.

### Treatment protocols and procedures

ILAT was shaped according to everyday request communication and related social interaction. Three patients and a therapist engaged in so-called “language games,” where players use verbal utterances to obtain picture cards from each other (cf. Difrancesco et al., 2012). Naming Therapy was conceived to resemble the group context of ILAT in as many ways as possible, except for the fact that participants did not use verbal utterances for communication and social interaction. Instead, the goal was to name or describe objects shown on the picture cards. Card sets were counterbalanced across treatment groups, with target words (*n* = 288 different pictures) and carrier phrases (e.g., “Give me the […]” in ILAT vs. “This is a […]” in Naming Therapy) tailored to the patients' individual language skills. Both types of training were delivered with the same high intensity (3.5 h per therapy session) and duration (six consecutive working days), resulting in overall 42 h of treatment within less than 4 weeks. The schedules included a 6-day recreation interval between the two treatments. None of the patients attended any other form of SLT throughout the entire trial. A clinical neuropsychologist tested each patient 1 day before (T_1_) and 1 day after the first training period (T_2_), as well as 1 day after the second training period (T_3_). The neuropsychologist was blinded to the group assignment and did not have patient contact apart from the testing sessions (for further details of the treatment protocols and procedures, see Stahl et al., 2016).

### Primary outcome measure

Changes in language abilities were assessed using a newly developed aphasia test battery, the Action Communication Test (ACT). This battery was designed to directly compare the practicability of utterance-centered and dialogue-sensitive outcome measures in SLT. In step one of the procedure (subscale ACT Naming), sets of five real generic objects were presented on a table (e.g., a flower, a bottle, a necklace, a key, and a thread). The patient was asked to name each of these objects, one by one. If the patient named an object correctly, the experimenter subsequently removed it from the table. If the patient failed to name an object twice, the experimenter removed this item, after ensuring via pointing that it was the intended one, and placed it in a bag. Target utterances were always preceded by a standardized question (experimenter: “What do you *see*?”). In step two of the procedure (subscale ACT Requests), the patient verbally requested sets of five objects presented on the table, again one by one. Whenever utterances were correct, the experimenter handed over the requested object to the patient who, eventually, placed it in a bag. After two failed attempts to make a request, the experimenter ensured via pointing that the patient received the intended object. As during step one, each target utterance was preceded by a standardized question (experimenter: “What do you *want*?”). Moreover, the subscale ACT Requests encouraged the use of formulaic expressions when handing over objects to the patient (e.g., “Here you are,” “Thank you” and “You're welcome”). This linguistic category of utterances is often preserved in aphasic speech and may be viewed as a motivational resource in diagnostic sessions (cf. Stahl and Van Lancker Sidtis, 2015; for examples illustrating the dialogue-sensitive nature of the ACT, see Table 3).

**Table 3:** **Dialogue-sensitive character of the ACT**.

**Speaker**	**Subscale ACT Naming**	**Subscale ACT Requests**	**Scoring (points)**
Experimenter	“What do you see?”	“What do you want?”	—
Patient	“A flower.”	“The mirror.”	“Flower” (2); “Mirror” (2)
Experimenter	[Takes the flower and places it in a bag.]	[Hands over the mirror.] “Here you are.”	—
Patient	—	[Places the mirror in a bag.] “Thank you.”	—
Experimenter	“What else do you see?”	What else do you want?”	—
Patient	“A cup, no …a bottle.”	“The life, no …the knife.”	“Bottle” (1); “Knife” (1)
Experimenter	[Takes the bottle and places it in a bag.]	[Hands over the knife.] “Here you are.”	—
Patient	—	[Places the knife in a bag.] “Thank you.”	—
Experimenter	“What else do you see?”	“You're welcome. What else do you want?”	—
Patient	“A…I don't know.”	“The…I don't know.”	“…” (0); “…” (0)
Experimenter	[Points to the necklace to ensure that this is the intended object.]	[Points to the ring to ensure that this is the intended object.]	—
Patient	[Gives some verbal or gestural sign of agreement.]	[Gives some verbal or gestural sign of agreement.]	—
Experimenter	[Takes the necklace and places it in a bag.]	[Hands over the ring.] “Here you are.”	—
Patient	—	[Places the ring in a bag.] “Thank you.”	—
Experimenter	“What else do you see?”	“What else do you want?”	—
Patient	[…]	[…]	[…]

*Turn-taking structure and scoring procedure of the Action Communication Test (ACT; from top to bottom). Each ACT subscale involves standardized questions (“What do you see?” or “What do you want?”), target utterances and, if necessary, verbal or gestural signs of agreement. The subscale ACT Requests also encourages the use of formulaic expressions (e.g., “Here you are,” “Thank you” and “You're welcome”; cf. Stahl and Van Lancker Sidtis, 2015)*.

Testing materials of the ACT consisted of 40 standardized objects that were allocated to two parallel lists (List A and List B). Each list included four sets of five items. The composition of these different sets did not change throughout the testing sessions. In both steps of the ACT, patients could freely choose the sequence of objects per set to be named or requested. Notably, even severely affected patients did not have problems to understand these procedures. Items of List A and List B were matched for a variety of psycholinguistic features, such as mean normalized lemma frequency, as well as the average number of syllables, phonetic sounds and consonant clusters at word onset. To prevent any item-specific influences, we used List A and List B in counterbalanced order across ACT subscales and treatment groups (for the complete inventory of objects and controlled psycholinguistic features, see Table 4). The scoring system of the ACT was as follows: two points for correctly produced target words; one point for correctly produced target words on the second attempt or incorrect, but semantically or phonologically related utterances (e.g., “cup” instead of “bottle” or “life” instead of “knife”); no points for any further utterances or omissions. Based on these ratings, the average total number of points obtained on the subscales ACT Naming and ACT Requests were expressed as normally distributed *t*-scores (with reference to language performance at T_1_). The combined *t*-scores on the subscales ACT Naming and ACT Requests served as primary outcome measure (Composite ACT).^2^ Additional analyses focused on the two ACT subscales separately from each other. Testing duration ranged from 10 to 20 min.

**Table 4:** **Two parallel lists of ACT items**.

	**List A**	**List B**
Set 1	Flower [Blume]	Mirror [Spiegel]
	Bottle [Flasche]	Knife [Messer]
	Necklace [Kette]	Ring [Ring]
	Key [Schlüssel]	Feather [Feder]
	Thread [Faden]	Bowl [Schale]
Set 2	Bell [Glocke]	Ball [Ball]
	Nail [Nagel]	Cup [Tasse]
	Fork [Gabel]	Brush [Pinsel]
	Button [Knopf]	Pipe [Pfeife]
	Goblet [Becher]	Hammer [Hammer]
Set 3	Pencil [Stift]	Glasses [Brille]
	Hook [Haken]	Spoon [Löffel]
	Can [Dose]	Stamp [Stempel]
	Comb [Kamm]	Syringe [Spritze]
	Screw [Schraube]	Saw [Säge]
Set 4	Pliers [Zange]	Tea Pot [Kanne]
	Perfume [Parfüm]	Scissors [Schere]
	Magnet [Magnet]	Sieve [Sieb]
	Alarm Clock [Wecker]	Compass [Kompass]
	Ointment [Salbe]	Pencil Sharpener [Spitzer]
Mean normalized lemma frequency (SD)	13.3 (11.7)	13.3 (10.3)
Average number of syllables (SD)	1.9 (0.4)	1.9 (0.4)
Average number of phonetic sounds (SD)	5.0 (0.7)	5.0 (1.3)
Average number of consonant clusters at word onset (SD)	1.4 (0.5)	1.4 (0.6)

*Two parallel lists of Action Communication Test (ACT) items, translated from German. Each word represents a real generic object used throughout all testing sessions. Original words consist of one or two syllables; compounds were excluded. Items are sorted by normalized lemma frequency (number of occurrences per million words) in descending order (from top to bottom), with values retrieved from the German dlex database (www.dlexdb.de). Note: To improve the psychometric quality of the ACT, we increased the number of sets per item list in subsequent research*.

*SD, Standard deviation*.

### Secondary outcome measure

For correlation analyses between the Composite ACT and an external criterion, language assessment also included an established aphasia test battery, known for its good construct validity, re-test reliability and suitability to interpret individual numerical changes over time (AAT; Huber et al., 1984). Language performance was measured on four AAT subscales: Token Test, Repetition, Naming, and Comprehension. We excluded the AAT subscales Spontaneous Speech (due to its insufficient construct validity) and Writing (considering the emphasis on spoken language in our treatment). Again, results were expressed as normally distributed *t*-scores, averaged across the four AAT subscales. Testing duration ranged from 60 to 90 min. Overlap between therapy materials and target utterances of both outcome measures was small (~5%) and varied with symptom severity, as patients with global aphasia and severe Broca's aphasia are typically trained with a limited selection of high-frequency items, whereas patients with mild-to-moderate Broca's aphasia benefit from a larger repertoire of card sets. This fact rules out the possibility to contrast trained and untrained items in the current RCT.

### Statistical analyses

Mann-Whitney *U* tests suggested that the two parallel lists of ACT items were well matched, as average scores did not differ significantly between List A and List B at any point in time (*z* <0.58, *p* ≥ 0.62, always not significant [n.s.]). Further Mann-Whitney *U* tests confirmed that Group I and Group II did not differ significantly with regard to their performances on the ACT (*z* = −0.39, *p* = 0.76, n.s.) or on the AAT (*z* = −0.65, *p* = 0.57, n.s.) at baseline (T_1_). For each outcome measure, a repeated-measures analysis of variance (ANOVA) was conducted, including within-subject factor Time (T_1_; T_2_; T_3_) and between-subject factor Group (Group I; Group II). Wilcoxon signed-rank tests were used for planned comparisons, and Kendall's τ for correlation analyses between the Composite ACT and AAT results. Preference was given to non-parametric methods, whenever possible, to account for the small sample size. A *post-hoc* repeated-measures ANOVA investigated the interaction of Time (T_1_; T_2_; T_3_), ACT Subscale (ACT Naming; ACT Requests) and Group (Group I; Group II). For all statistical analyses, two-tailed *p*-values and alpha levels of 0.05 were applied.

## Results

**Figure 2:**
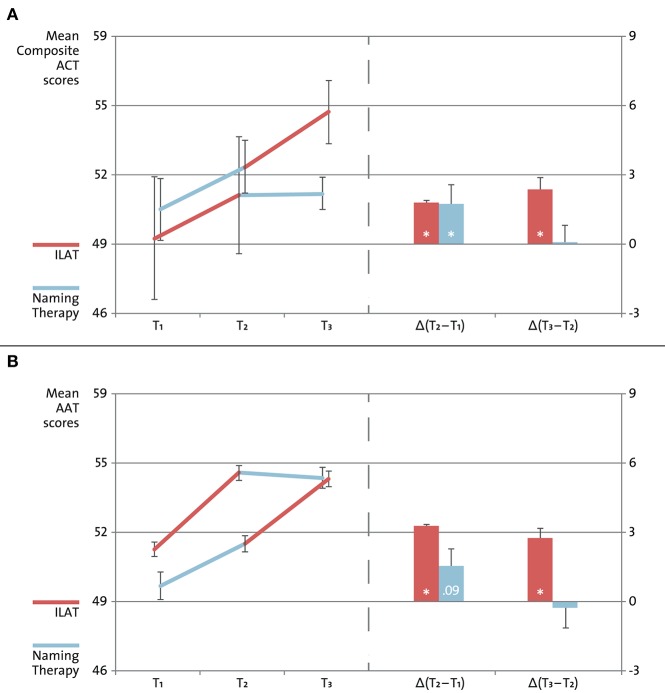
**Aphasia test results**. Changes in language performance on the Action Communication Test (ACT; **A**) and on the Aachen Aphasia Test (AAT; **B**). Fourteen individuals with post-stroke chronic non-fluent aphasia were randomly assigned to one of two treatment groups: Intensive Language-Action Therapy (ILAT; shown in *red*) administered prior to Naming Therapy (shown in *blue*), or vice versa. Patients were tested at three points in time: before treatment onset (T_1_), after the first treatment (T_2_), and after the second treatment (T_3_). Scores on the ACT and AAT revealed similar patterns of changes in language performance in the first training period [Δ(T_2_–T_1_)] and in the second training period [Δ(T_3_–T_2_)], as indicated by Wilcoxon signed-rank tests (*p <0.05). Mann-Whitney *U* tests confirmed that Group I and Group II did not differ significantly with regard to their performances on the ACT (*p* = 0.76) or on the AAT (*p* = 0.57) at baseline (T_1_).

A repeated-measures ANOVA revealed a significant interaction of Time and Group based on the Composite ACT scores [*F*_(2, 24)_ = 3.90, *p* = 0.03, η^2^ = 0.10]. In the *first* training period, Wilcoxon signed-rank tests suggested significantly increased scores on the Composite ACT with ILAT (*z* = 2.21, *p* = 0.03) and with Naming Therapy (*z* = 2.12, *p* = 0.03). In the *second* training period, ILAT alone was found to be effective (*z* = 2.51, *p* = 0.01), in contrast to Naming Therapy (*z* = 0.27, n.s.; see Figure 2A and Table 5). Additional analyses addressed language performance on the two ACT subscales. A repeated-measures ANOVA replicated the interaction of Time and Group on the subscale ACT Requests [*F*_(2, 24)_ = 3.69, *p* = 0.04, η^2^ = 0.11], but not on the subscale ACT Naming [*F*_(2, 24)_ = 1.83, n.s.]. Exploring this potential difference in utterance-centered and dialogue-sensitive speech-language testing, a *post-hoc* repeated-measures ANOVA showed a positive trend for the interaction of Time, ACT Subscale and Group [*F*_(2, 24)_ = 3.09, *p* = 0.06, η^2^ = 0.05].

**Table 5:** **Aphasia test scores**.

	**T_1_**	**T_2_**	**T_3_**	**Δ(T_2_–T_1_)**	**Δ(T_3_–T_2_)**
**Composite ACT**					
Group I (SD)	49.2 (12.7)	51.0 (12.8)	51.1 (13.5)	1.8 (0.6)^*^	0.1 (1.1)
Group II (SD)	50.6 (8.4)	52.3 (8.4)	54.5 (7.2)	1.8 (2.3)^*^	2.2 (1.5)^*^
**ACT Naming**					
Group I (SD)	49.1 (12.2)	51.1 (12.4)	51.0 (12.8)	2.1 (1.3)^*^	−0.1 (1.1)
Group II (SD)	50.7 (8.9)	52.0 (7.8)	54.0 (8.0)	1.3 (3.4)	2.1 (1.4)^*^
**ACT Requests**					
Group I (SD)	49.5 (12.9)	50.9 (13.0)	51.2 (14.0)	1.5 (1.3)^*^	0.2 (1.4)
Group II (SD)	50.4 (8.1)	52.6 (8.8)	55.0 (6.3)	2.2 (1.6)^*^	2.3 (2.9)^*^
**AAT**					
Group I (SD)	50.8 (9.7)	53.8 (9.6)	53.2 (10.6)	3.3 (0.3)^*^	−0.4 (2.0)
Group II (SD)	48.7 (6.7)	50.3 (7.0)	52.9 (7.5)	1.6 (2.2)	2.7 (1.6)^*^

Mean t-scores obtained on the Action Communication Test (ACT; see Materials and Methods) and on the Aachen Aphasia Test (AAT; Huber et al., 1984). Fourteen patients with post-stroke chronic non-fluent aphasia were randomly assigned to one of two treatment orders: Intensive Language-Action Therapy administered prior to Naming Therapy (Group I), or vice versa (Group II). The patients were tested at three points in time: before treatment (T_1_), after the first treatment (T_2_), and after the second treatment (T_3_). Asterisks refer to significantly improved language performance in the first training period [Δ(T_2_–T_1_)] and in the second training period [Δ(T_3_–T_2_)], as revealed by Wilcoxon signed-rank tests (

* *p <0.05)*.

*SD: Standard deviation*.

Focusing on the AAT results (averaged across four selected subscales, as specified above), a repeated-measures ANOVA confirmed the interaction of Time and Group [*F*_(2, 24)_ = 4.37, *p* = 0.02, η^2^ = 0.10]. Consistent with the Composite ACT scores, Wilcoxon signed-rank tests indicated similar patterns of changes in language performance on the AAT. In the *first* training period, we observed significant progress with ILAT (*z* = 2.21, *p* = 0.03) and a positive trend with Naming Therapy (*z* = 1.70, *p* = 0.09). Once more, only patients receiving ILAT continued to make progress in the *second* training period (*z* = 2.37, *p* = 0.02), while patients receiving Naming Therapy did not (*z* = 0.11, n.s.; see Figure 2B and Table 5). Correlations between the Composite ACT and the AAT were large at each point in time (T_1_, T_2_, and T_3_: Kendall's τ = 0.66, 0.82, and 0.83; always *p* ≤ 0.001; overall explained common variance: 60%). The achieved statistical power exceeded the critical threshold of 95% on both outcome measures (calculations with number of groups: 2; number of repeated testing sessions: 3; Cohen's *f* ≥ 0.5 derived from partial η^2^ ≥ 0.21 in our patient sample, congruent with effect sizes reported in Stahl et al., 2016; resulting in 1–β ≥ 0.98; cf. Faul et al., 2009).

## Discussion

The present study aimed to compare the suitability of utterance-centered and dialogue-sensitive outcome measures in speech-language testing. Fourteen individuals with post-stroke chronic non-fluent aphasia each received two types of intensive training in counterbalanced order: conventional confrontation naming (Naming Therapy), and communicative-pragmatic SLT (ILAT). Both types of training were delivered with the same high intensity and duration, with therapy materials and number of utterances carefully matched between treatment groups. Results on the Composite ACT and the AAT revealed similar patterns of changes in language performance over time: ILAT proved to be effective, regardless of when this method was administered, whereas Naming Therapy led to significant increases (Composite ACT) or a positive trend (AAT) in aphasia test scores only when given at the onset of the treatment. Changes in language performance were relatively consistent with the AAT results also when considering both ACT subscales separately from each other. Taken together, these promising findings emphasize the need for further studies to confirm the psychometric properties of the ACT in an extended patient sample.

Although we acknowledge the slightly elevated risk of false-positive results arising from multiple comparisons, we wish to highlight that correlations between the Composite ACT and the AAT were large at each point in time, ranging from 0.66 to 0.83 (always *p* ≤ 0.001). These correlations may reflect the strong congruence between our two outcome measures over and above the utterance-centered or communicative-pragmatic character of the training, indicating a possible general adequacy of the ACT in evaluating treatment-induced progress. Overall, the Composite ACT and AAT scores shared 60% of the variance explained by changes in language performance in our data. However, testing duration was substantially shorter for the ACT (10–20 min) than for the AAT (60–90 min). While traditional aphasia test batteries are likely to be more accurate in documenting isolated skills in verbal expression and comprehension, our findings suggest that the Composite ACT may be equally sensitive and more time-effective in assessing the efficacy of SLT.

A main motivation for using dialogue-sensitive diagnostic instruments in aphasia therapy comes from linguistic-pragmatic theory and from neuroscience data. A number of studies indeed show an increase of neural activity associated with verbal requests compared to object naming (Egorova et al., 2013, 2014, 2016) and a close functional relationship between cortical language and motor regions (Pulvermüller et al., 2005; Glenberg et al., 2008; Kemmerer et al., 2008; Willems et al., 2011; Andres et al., 2015). One may therefore argue that embedding language in communication and social interaction potentially leads to synergies in left perisylvian eloquent areas (Berthier and Pulvermüller, 2011). Such synergies might enhance the quality of speech-language testing if verbal utterances are grounded in the rich action-sequence structure known from everyday communication. This claim is consistent with the fact that our dialogue-sensitive approach evaluating verbal requests tended to be more successful in distinguishing between different types of training in our patient sample than utterance-centered object naming (ANOVA interaction of Time, ACT Subscale and Group: *p* = 0.06).

The present RCT provides preliminary, yet encouraging evidence that a recently developed diagnostic instrument, the ACT, is both sensitive and time-effective in assessing the outcome of SLT. Future research will be needed to substantiate these findings. We wish to note that, compared to the AAT results, variability in language performance was higher on the Composite ACT and its two subscales (e.g., standard deviation of Group II on the subscale ACT Naming between T_1_ and T_2_: 3.4; on the subscale ACT Requests between T_2_ and T_3_: 2.9; see Table 5). More items per subscale are likely to produce smaller variability measures that, in turn, may help improve the statistical power of the ACT, observe cross-sectional differences in verbal expression depending on naming or request tasks, and detect individual longitudinal changes above chance level in diagnostic sessions. A subsequent RCT is currently underway, exploring the construct validity and re-test reliability of the ACT with expanded sets of items in a larger patient sample. Moreover, we are collecting normative data from healthy age-matched controls alongside persons with chronic post-stroke aphasia to determine the suitability of the ACT in identifying speech-language pathologies. We hope that these studies will eventually establish our new method for application in clinical practice.

## Author contributions

Significant contributions include: study concept and design (BS, BM, and FP), treatment protocols and materials (BS, BM, and FP), trial coordination and therapy sessions (BS), testing sessions (BM), structural magnetic resonance imaging and lesion overlay maps (FD and GL), statistical analyses (BS), data interpretation (BS, BM, FD, GL, and FP), manuscript drafting and artwork (BS), and revisions (BS, BM, FD, GL, and FP).

## Funding

This research was supported by Deutsche Forschungsgemeinschaft (Pu 97/15-1 to FP) and Deutscher Akademischer Austauschdienst (fellowship to GL).

### Conflict of interest statement

The authors declare that the research was conducted in the absence of any commercial or financial relationships that could be construed as a potential conflict of interest.

## Footnotes

^1^For simple logistic reasons, it was not possible to administer the current set of aphasia test batteries in the complete patient sample reported in Stahl et al. (2016).

^2^The presentation of our two outcome measures differs from their order of appearance in the trial registration. For didactic purposes, we here defined our major dependent variable, the ACT, as “primary” outcome measure, while our external criterion, the AAT, served as “secondary” outcome measure.
